# Patient preferences for epilepsy treatment: a systematic review of discrete choice experimental studies

**DOI:** 10.1186/s13561-023-00431-0

**Published:** 2023-03-18

**Authors:** Sinaa Al-Aqeel, Reem Alotaiwi, Bushra Albugami

**Affiliations:** Clinical Pharmacy Department, College of Pharmacy, King Saud University, Riyadh, Saudi Arabia

**Keywords:** Patient preferences, Systematic review, Discrete choice experiments, Epilepsy

## Abstract

**Background:**

This review aimed to 1) identify and assess the quality of discrete choice experiments (DCEs) examining preferences related to epilepsy treatment; 2) summarize the attributes and attribute levels measured in these studies; 3) identify how researchers selected and developed these attributes; and 4) identify which attributes are most important for epilepsy patients.

**Methods:**

A systematic literature review using PubMed, Web of Science and Scopus databases from database inception to February or April 2022. We included primary discrete-choice experiments eliciting preferences for various attributes of pharmacological and surgical interventions in patients diagnosed with epilepsy or the parents/carers of children with epilepsy. We excluded non- primary studies, studies assessing preferences for nonpharmacological treatment and studies that elicit preferences using methods other than discrete choice experiments. Two authors independently selected studies, extracted data and assessed risk of bias of studies. The quality of the included studies was assessed using two validated checklists. Study characteristics and findings were summarized descriptively.

**Results:**

A total of seven studies were included in the review. The majority of studies explored patients’ preferences, and two compared the preferences of patients with physicians. The majority (*n* = 6) compared two medications, and one compared two surgical options to continuing medication options. The studies examined 44 attributes in total, including side effects (*n* = 26), efficacy expressed as being seizure free or have fewer seizures (*n* = 8), costs (*n* = 3), dosing frequency (*n* = 3), duration of side effects (*n* = 2), mortality (*n* = 1), long-term problems after surgery (*n* = 1) and surgical options (*n* = 1). The findings indicate that people with epilepsy have strong preferences for improving seizure control, which was ranked as the top priority in all studies. Patients also have a strong preference for the reduction of adverse effects and may be willing to make trade-offs between improved seizure control and reduction of long-term side effects that may impact their quality of life.

**Conclusions:**

The use of DCEs in measuring patients’ preference for epilepsy treatment is accumulating. However, inadequate reporting of methodological details may reduce decision-makers’ confidence in the findings. Suggestions for future research are provided.

**Supplementary Information:**

The online version contains supplementary material available at 10.1186/s13561-023-00431-0.

## Background

Epilepsy is a common neurological disease affecting approximately 70 million people worldwide [1]. Patients with epilepsy start with a single medication (monotherapy), and if monotherapy is ineffective, an add-on treatment to the primary medication is considered. The selection of the medication depends on many factors, including seizure type, risk and benefit of medications and age of the patient.

The move to patient empowerment has been conveyed in the incorporation of patients’ values in decision-making [2]. This emphasized patients’ participation at different levels, including setting research priorities [3], developing clinical guidelines [4, 5] and supporting reimbursement decisions [6]. One example of incorporating patient preferences into clinical guidelines is the National Institute for Health and Care Excellence guidelines [7], which recommend that the treatment plan with antiseizure medications in children, young people and adults with epilepsy should take into account clinical factors as well as “the preferences of the person, and their family or carers if appropriate”. The differences in efficacy and safety profile of antiepileptic drugs signify the importance of assessing patient preferences. Studying patient preferences could help identify important clinical endpoints, relative importance of treatment characteristics and acceptability of risk–benefit trade off [8]. Potential benefits for incorporating patient preferences in healthcare decision making include improved adherence, effectiveness and outcomes of an intervention [8, 9].

There are two approaches to measuring preferences: revealed and stated [10]. The stated preference methods are a common approach in the health economics literature**.** Ryan et al. [11] review classified the methods for eliciting public preferences for health care into quantitative methods, including ranking, rating and choice-based approaches, and qualitative methods, including interviews, the Delphi technique, focus groups and consensus panels. In a more recent review and taxonomy of preference methods, Soekhai et al. [12] define preference elicitation methods as quantitative methods for collecting quantifiable data for hypothesis testing and other statistical analyses, including discrete choice-based methods, ranking methods, indifference methods and rating methods. Qualitative methods that collect descriptive data through participant or phenomenon observation and examine the subjective experiences and decisions made by participants through interviews, focus groups, and meetings are grouped under preference exploration methods [12].

A discrete choice experiment (DCE) is an example of choice-based techniques commonly used to elicit a preference for health care interventions, services or policies. In a DCE, respondents are asked to choose between two or more alternatives characterized using different attributes. Each attribute has different levels. Different DCE tasks can be assembled by changing combinations of attribute levels. It is assumed that respondents select the alternative that provides the greatest utility to them. DCEs are based on random utility theory and are consistent with economic demand theory [13, 14]. The terms discrete choice experiment and conjoint analysis are used interchangeably in the literature, although they are not synonymous [13].

With the increased number of publications examining patient preference for treatment using the DCE approach, a number of systematic reviews have examined the quality of these studies and synthesized their findings on the important attributes of patient preferences for the treatment of diseases such as anxiety and depressive disorders [15], cancer [16] and asthma [17]. Systematic reviews collate current evidence and indicate where specific gaps in knowledge exist to identify priorities for further research. This review aimed to 1) identify and assess the quality of discrete choice experiments examining preferences related to epilepsy treatment; 2) summarize the attributes and attribute levels measured in these studies; 3) identify how researchers selected and developed these attributes; and 4) identify which attributes are most important for patients with epilepsy.

## Methods

The review protocol was registered in the International Prospective Register of Systematic Reviews (PROSPERO) database (CRD42022309430).

The study is reported in accordance with the Preferred Reporting Items for Systematic Reviews and Meta-analyses (PRISMA) guidelines [18].

### Eligibility criteria

We included primary studies using discrete-choice experiments to elicit preferences for various attributes of pharmacological and surgical interventions in patients diagnosed with epilepsy or the parents/carers of children with epilepsy.

The following types of studies were excluded:

- Primary studies measuring stated preferences for nonpharmacological treatment, such as vagal nerve stimulation and ketogenic diet.
- Primary studies eliciting preferences using methods other than discrete choice experiments such as methods used to estimate the monetary values of a product or service (e.g., contingent valuation or willingness-to-pay methods) and methods used to generate utility weight for quality-adjusted life years (QALYs) calculation (e.g. time-trade-off or standard gamble).
- Nonprimary studies (e.g., review studies, commentaries and editorials)
- Studies published only as abstracts or conference proceedings.
- Studies published in languages other than English.

### Information sources

We searched the PubMed, Web of Science and Scopus databases from inception until the date of the search (February 2022 or April 2022). The reference lists of the included articles were reviewed for potential studies.

### Search strategy

Search terms for stated preferences derived from previous systematic reviews on DCEs and recommended searching strategies [19, 20] for preference studies were used. This included words such as “conjoint analysis”, “conjoint choice experiment”, and “stated preference”. These terms were combined with subject headings and free text terms for epilepsy. The specific terms used to search each database and search date are available in Appendix 1.

### Selection process

Three reviewers screened the titles and abstracts. First, two reviewers (RA, BA) independently screened the titles and abstracts. A third unblinded reviewer (SA) screened all titles and abstracts, made decisions and identified disagreements between the reviewers. Disagreements regarding study inclusion or exclusion were resolved by discussion. The Ryaan tool was used for title and abstract screening and selection.

### Data collection process

Data were extracted by one reviewer (RA, BA) and reviewed for accuracy and completeness by a second reviewer (SA). Excel was used to collect data.

### Data items

We collected data on study characteristics (e.g., first author, year of publication, study objective, country, sample size, main results), attributes and levels of information (e.g., attribute and level identification, selection and labeling, the mode of survey administration, relative importance of each attribute category).

### Study risk of bias assessment

The risk of bias assessment was assessed using the PREFS (Purpose, Respondents, Explanation, Findings, Significance) checklist [21] and ISPOR Conjoint Analysis Applications in Health Checklist [10], a 10-item checklist. Two independent reviewers carried out risk of bias assessment, and disagreements were resolved by discussion.

### Synthesis methods

A descriptive analysis of the data in a narrative format, accompanied by tables to convey relevant study components, was conducted.

## Results

### Study selection

**Fig. 1:**
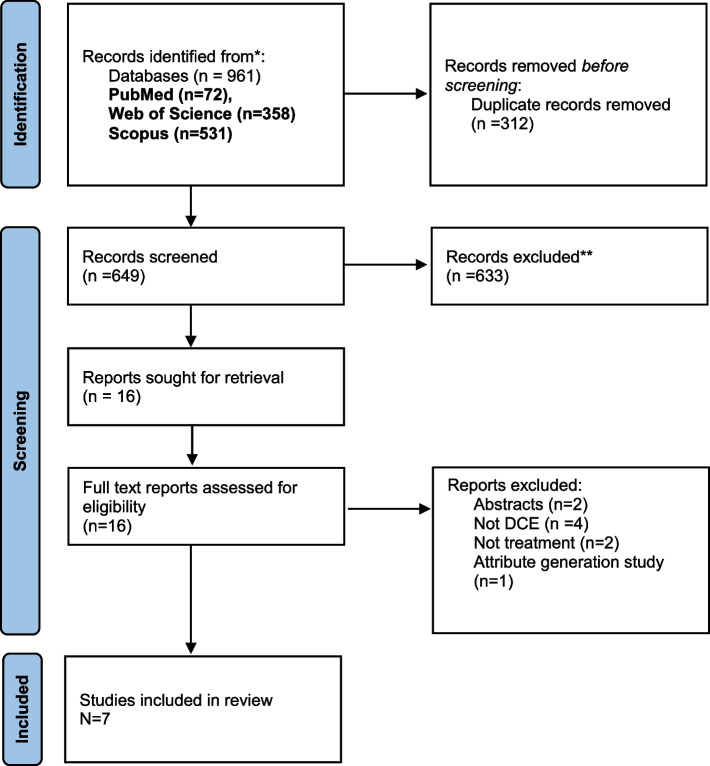
Flow diagram of study selection

The search identified 961 records. After removing duplicate records (*n* = 312), we screened the titles and abstracts and excluded 633 articles. At this stage, the main reasons for exclusion were not DCE (*n* = 617), DCE but not treatment (*n* = 11), and no stated preference (*n* = 5). The full text of 16 articles was assessed for eligibility; 7 were included [22–28], and 9 were excluded. Three titles were without abstracts, and after obtaining the full text were excluded because they were not DCEs. One study was not a DCE [29]. One study was a DCE of self-management programs [30]. One discrete choice study that compared society and patients’ preferences for health priorities and epilepsy patients was 2% of their sample [31]. One study was excluded because it was a focus group of physicians and caregivers to identify attributes for future discrete choice experiments [32]. Two abstracts were excluded. We contacted the authors of these abstracts to enquire if they were published in full. One abstract was not published [33], the other abstract was published, and the full text was excluded for the abovementioned reason [32]. Figure 1 shows the flow diagram of the included studies and the reasons for exclusion.

### Study characteristics

The majority of the studies were published after 2017 (*n* = 5). Five studies explored patients’ preferences, and two compared the preferences of patients with those of physicians [22, 27]. Six studies compared two medications, and one compared two surgical options to continuing medication options [28]. The online survey was the most common mode of administration (*n* = 6). The questionnaire was mainly self-administered by participants (*n* = 6). Three studies were from the US, two from the UK, one from China, and one with participants from different European countries. The sample size ranged from 148 to 518 patients (Table 1). The studies were published in clinical journals, with only one published in a specialized pharmacoeconomic journal [25]. Three studies [22, 25, 27] were funded by pharmaceutical companies.

**Table 1:** Study characteristics

Author, Year, Country	Study aim [sample size]	Attributes identification and selection	Attributes [levels]
Lloyd, 2005, UK [25]	To elicit the preferences of people with epilepsy for changes in adverse effects and the level of seizure control, and to determine willingness to pay for improvements in adverse effects and seizure frequency [148]	Literature review, drug monographs and discussion with epilepsy advocacy group	1. Seizure control [you become free of seizures, your seizures are reduced by more than half (75%), or your seizures are reduced by half (50%)] 2. Weight change [you gain 26 lbs, you gain 13 lbs, or you lose 13 lbs] 3. Skin rash [you have no skin rash, you have a 1 in 10 chance (10%) of developing skin rash, or you have a 1 in 5 chance (20%) of developing skin rash] 4. Concentration [your concentration is unaffected, you have a 1 in 10 chance (10%) of the drug affecting your concentration, or you have a 1 in 5 chance (20%) of the drug affecting your concentration] 5. Alopecia [you have no hair loss, you have a 1 in 10 chance (10%) of hair loss, or you have a 1 in 5 chance (20%) of hair loss] 6. Feeling sick [you do not feel sick, you have a 1 in 10 (10%) chance of feeling sick or being sick, or you have a 1 in 3 (33%) chance of feeling sick or being sick] 7. Payment vehicle cost [the drug will cost you an extra £25 per month, the drug will cost you an extra £50 per month, or the drug will cost you an extra £75 per month]
Manjunath, 2012, USA [26]	To quantify the relative importance of treatment outcomes of add-on AEDs and the extent to which patients with symptoms of partial seizures were willing to accept short-term and long-term side effects in exchange for better seizure control and estimate patients' WTP for treatment outcomes [193]	Literature review, consultation with epileptologists and patient interviews	1. Number of seizures [zero seizures, 75% fewer seizures, 50% fewer seizures, and 25% fewer seizures] 2. Duration of short-term effects [none—last for 1 week, and last for 4 weeks] 3. Long-term fatigue or moodiness [none—cause some difficulty doing daily activities] 4. Long-term confusion or memory problems [none—cause some difficulty doing daily activities] 5. Difficulty urinating [none, half the time, and almost always] 6. Weight change in 6 months [15 pound weight loss, no weight loss or gain, and 15 pound weight gain] 7. How often you take the medicine [once a day, twice a day, and 3 times a day] 8. Personal medicine cost [low cost range [$0 per month ($0 every 3 months), $25 per month ($75 every 3 months), $75 per month ($225 every 3 months) $150 per month ($450 every 3 months)] – [high cost range [$0 per month ($0 every 3 months), $75 per month ($225 every 3 months), $150 per month ($450 every 3 months), $300 per month ($900 every 3 months)]]
Ettinger, 2018, USA [22]	To determine the relative importance of AED attributes to treatment preference among both patients and neurologists, and the level of concordance and discordance between the two groups of participants. regarding AED attributes for treating epilepsy [518 patients and 202 neurologists]	Literature review, consultation with 4 neurologist with experience researching and/or treating epilepsy	1. AED efficacy [completely seizure-free, seizures reduced by more than half, seizures reduced by a half, and seizures reduced by less than half] 2. Dosing frequency [one time per day, two times per day, three times per day, four times per day, and five or more times per day] 3. Coordination/balance side effects [0% chance of problems, 5% chance of problems, 10% chance of problems, 15% chance of problems, and 20% chance of problems] 4. Psychiatric side effects [0% chance of problems, 5% chance of problems, 10% chance of problems, 15% chance of problems, and 20% chance of problems] 5. Diminished energy level [0% chance of problems, 5% chance of problems, 10% chance of problems, 15% chance of problems, and 20% chance of problems] 6. Dietary restrictions [can swallow, crush, mix with food—cannot swallow, crush, mix with food]
Holmes, 2019, UK [23]	To compare patient-defined benefit-risk preferences with the results of a clinical trial of AEDs and to assess differences between patient subgroups [280: 177 patients with a recent or established diagnosis, 103 women of childbearing potential]	Qualitative study of epilepsy patients to select important outcomes derived from clinical trials and validated outcome measures and nominated by the patients. The validity of top 10 outcomes were examined using cognitive interviews with patients and a meeting with prescribing physicians. The top 5 attributes were used	1. Seizures stop [5 in 10 people -0.5- or 3 in 10 people -0.3-] 2. Fewer seizures [3 in 10 people -0.3- or 1 in 10 people -0.1-] 3. Memory problems [1 in 100 people -0.01- or 7 in 100 people -0.07-] 4. Depression [1 in 100 people -0.01- or 8 in 100 people -0.08-] 5. Feelings of aggression [1 in 100 people -0.01- or 8 in 100 people -0.08-] 6. Harm to your fetus if the patient gets pregnant while taking this medication [2 in 100 pregnant women -0.02- or 9 in 100 pregnant women -0.09-]
Hua, 2020, China [24]	To explore Chinese patients’ risk–benefit preferences and willingness-to-pay for AEDs treatment [151]	Literature review, calibrated by neurologists and patient survey	1. Efficacy of AED [seizure-free -100% controlled-, seizure frequency is reduced by more than half -75% less-, or seizure frequency is reduced by half -50% less-] 2. Digestive adverse reaction [0 in 10 people -0%-, 1 in 10 people -10%-, or 3 in 10 people -30%-] 3. Duration of side effects in neuropsychic systems [none, last for 1 week, or last for 4 weeks) 4. Dosing frequency [once a day, twice a day, or thrice a day] 5. Effects of AEDs on the fetus [3 in 100 people -3%-, 6 in 100 people -6%-, or 10 in 100 people -10%-], 6. Drug costs [200¥ per month, 400¥ per month, or 600¥ per month]
Sinha, 2022, USA [28]	To determine patient acceptability of benefit-risk trade-offs in selecting treatment options for drug-resistant mesial temporal lobe epilepsy, including open brain surgery, laser ablation (laser interstitial thermal therapy [LITT]), and continued medications [406]	Consultation with an advisory group of four patients with drug-resistant epilepsy	1. Surgical options [Open brain surgery, Laser surgery] 2. Probability of being seizure free for at least 2 years [70%, 60%, 40%, 20%] 4. Probability of 30-day mortality [0%, 1%, 5%, 10%] 4. Probability of long-term problems after surgery [0%, 10%, 20%, 40%]
Rosenow, 2022, 8 European countries [27]	To elicit patients' preferences for attributes characterizing AEDs monotherapy options before treatment consultation, and to explore the trade-offs patients consider between treatment efficacy and risks of side effects. [404: 310 patients, 94 physicians]	Five experts—four physicians and one patient ambassador selected 7 attributes from a list of 15. The 15 attributes were selected by the funders’ staff from a list of 67 attributes. The source of the original 67 attributes was not explained	1. Chance of becoming seizure-free [60% chance, 45% chance, 25% chance] 2. Chance of developing clinical depression [None, 4% chance, 10% chance] 3. Personality changes [None, Mild, Moderate to severe] 4. Trouble thinking clearly [None, Mild, Moderate to severe] 5. Dizziness [None, Mild, Moderate to severe] 6. Change in body weight in 6 months [5% weight loss, No change, 5% weight gain] 7. Feeling sleepy or tired [None, Sleepiness, Mild-to-moderate tiredness, Severe tiredness]

### Risk of bias in studies

Using the PREFS checklist for assessing quality (Appendix 2), we identified one major shortcoming: the lack of reporting on the number of candidates approached and those who declined to participate and the differences between responders and nonresponders, if any. One study used a postal survey and reported the response rate to be 28% [25], and another study reported 33 patients who consented to participate in the study but did not complete the survey [23]. Both studies did not report any details of nonrespondents’ characteristics.

Appendix 2 presents the ISPOR risk of bias checklist for the included studies. The following section will discuss the risk of bias in the included studies in reference to the ISPOR checklist.

#### Research question

In all studies, the hypothesis, perspective and decision context of the analysis were implicit in the research question itself. Six studies examined patients’ trade-off between of antiepileptic drugs (AEDs) efficacy and side effects. One study [28] determined the tradeoffs of drug-resistant epilepsy patients between the risks and benefits of three options: surgery, laser ablation and continued treatment. Few studies have explored preferences further and compared preferences between different patient groups, such as women of childbearing potential and patients with recent diagnoses [23], between patients and physicians [22, 27] and before and after treatment consultation [27].

#### Attributes and levels

The studies examined 44 attributes in total (Table 1), including side effects (*n* = 26), efficacy expressed as seizure-free or fewer seizures (*n* = 8), costs (*n* = 3), dosing frequency (*n* = 3), duration of side effects (*n* = 2), mortality (*n* = 1), long-term problems after surgery (*n* = 1) and surgical options (*n* = 1). The types of side effects examined were weight change, harm to the fetus, depression, psychiatric side effects, feelings of aggression, personality changes, diminished energy level, feeling sleepy or tired, long-term fatigue or moodiness, skin rash, concentration, trouble thinking clearly, adverse digestive reaction, feeling sick, long-term confusion or memory problems, memory problems, difficulty urinating, coordination/balance side effects, dizziness, dietary restriction, probability of 30-day mortality and alopecia. The number of attributes per study was 6 (*n* = 3), 7 (*n* = 2), 4 (*n* = 1) and 8 (*n* = 1).

Four studies [22–25] reported literature as sources for the identification of attributes, one [28] reported consultation with an advisory group of four patients, one [26] reported literature of controlled trials, consultation with an epileptologist and patient interviews, and one [27] did not report the source of attribute identification (Table 1).

Although references for literature searched for attributes were provided, there were no details on the comprehensiveness of the literature search strategy. The type of study design used for attribute identification was reported by three studies as systematic reviews [25] and clinical trials [23, 26].

The selection of the attributes subset that was included in the analysis was achieved by consulting the epilepsy advisory group and a physician [25], prioritization by four neurologists [22], calibration by a neurologist [24], interviews with patients (*n* = 41) and physicians (*n* = 9) [23], and meeting with a group of four physicians and a patient representative [27]. Two studies mentioned no information on the selection of attributes [26, 28]. Only one [23] study provided a good description of the range of qualitative approaches used, such as semistructured interviews and think-aloud practices.

The level selection for each attribute was discussed by four studies and was from the literature [22, 24, 25], clinical trials [27] or clinical trials plus information on risk from an epilepsy charity website [23]. The levels were a mixture of categorial and probability (Table 1). The majority used 2–3 levels per attribute. There was no use of range except in one [27] study categorial level (mild to moderate). The cost attribute levels were captured from the UK price list in one study [25].

#### Construction of tasks

Although not explicitly reported that a full profile was used to construct the tasks, all studies presented alternatives with all the attributes that are being considered in the study rather than a subset of attributes (partial profile).

The format of the choice question was binary, where respondents were forced to choose between two alternatives in each set/task except in one [28] study where the status quo option of continuing with current medication was added to the two surgical profiles.

One [26] study included an optout option. The justification for not including an optout option was provided in one study [25].

#### Experimental design

The choice of experimental design to create a choice structure was reported in four studies as orthogonal balanced [25], Bayesian-optimized orthogonal design [22], D-optimal design [24], and D-efficient fractional-factorial experimental design [28]. One study [25] justified the choice of experimental design. The software used for data analysis was mentioned in all studies except one [27].

LIoyd et al. [25] presented a good explanation for the design diagnostics and evaluation, such as the number of overlapping attributes, level of balance and correlation among attributes, while reports in other studies were limited.

The number of choice tasks ranged from 8 to 18. Two studies reported using five [27] and twenty [28] versions of choice tasks. Homles et al. [23] reported using two versions of similar attributes except for the version for recently diagnosed patients included feelings of aggression, whereas women of childbearing age included the risk of fetal abnormality.

#### Preference elicitation

Three studies [23, 25, 28] reported clear information on the explanation of conjoint tasks to participants. Incentives were provided in one study [25].

In all studies, preferences were elicited with a discrete-choice design or forced choice format. Three studies [24–26] used preference estimates to calculate the willingness to pay for attributes.

One study [26] included an optout option as a separate question following each task.

First, the patients were asked to select their preferred add-on AED; then, they were asked whether they would add the preferred AED to their current treatment regimen.

In another study [28], the status-quo option of continuing on medication was included in the choice task.

Other qualifying questions, such as the strength of preferences, confidence in responses, and an estimate of their willingness to accept the less preferred option, were not reported.

#### Instrument design

All studies collected sociodemographic characteristics and health status information, such as the number of AEDs, type of epilepsy and seizure frequency.

All studies presented an example of a choice task in the manuscript. Some studies used pictograms to illustrate probabilities in choice tasks [23, 24, 27, 28], while others [25] used more than formats to present uncertainty. Three studies [23, 24, 28] reported descriptions of attributes in the manuscript. Two studies [25, 28] reported on the introductory section of the DCE instrument which explain attributes and levels and instructions on how to complete the choice task.

One [25] study reported that the scenarios were randomly paired up. No study reported that the task orders were randomized.

Two studies reported on pilot testing in a group of patients with sample sizes of 30 [25] and 10 [27] patients. Another study [23] reported piloting in a convenient sample of clinicians and staff. One study [22] reported pilot testing with four neurologists during the ranking process, and another study [28] reported a process to refine the survey involving using a read-aloud and think-aloud technique with patients. Two studies [24, 26] did not report any information on piloting.

#### Data collection

The sample size ranged from 148 to 518 patients. One study [23] reported sample size calculation, one [22] referred to similar published studies with similar sample size, two [24, 26] referred to a published reference to support their sample size and three [25, 27, 28] did not comment on sample size.

The recruitment of participants was through a research firm [22, 26, 28], an epilepsy charity [23, 25], an epilepsy center at a hospital [24] and part of a larger prospective study [27]. In one study [28], two cohorts were recruited from the study site clinic and the web passed panel.

The survey was administered via the mail [25], face to face [24] and online in five studies [22, 23, 26–28]. One study [27] specified that it was administered online at the study site. One study [25] reported that the questionnaire was posted to random members of the epilepsy group, while others [27, 28] reported that each participant was randomly assigned different versions of the questionnaires.

#### Statistical analyses

All studies collected respondents’ characteristics. Six [23–28] studies assessed the impact of sociodemographic and personal characteristics, such as seizure control, type of epilepsy, income, keeping seizure diaries, education, pregnancy intentions and previous surgery experience, on treatment preferences.

The quality of response was evaluated in five studies by including an option that was clearly superior to other scenarios [23–25] or identifying participants with no variation in their responses [26, 28]. Failing the quality response led to the exclusion of the respondents from the analysis in two [24, 26] studies. One [25] study reported the number of those who failed without exclusion, and another [23] reported the number of respondents who failed the question and that a model analysis excluding those patients was not significant.

One [25] study examined validity by exploring the variation in WTP based on individual income level and seizure frequency.

The model estimation was conducted using a random effects probit model [25], combined conditional logit and ranked logit model [26], hierarchical Bayes [22], random effects logit model [23], conditional logit model (McFadden’s choice model) [24] and random parameters logit [28]. One study [27] used a random parameters logit model for patients’ data and a conditional multinomial logit model for physicians’ data.

The statistical software reported were SAS, SPEED, GAUSS, Sawtooth and STATA.

#### Results and conclusions

The conclusions were supported by the study findings. All studies discussed the limitations of their findings.

The preference results were presented using a variety of methods, including preference weights [23, 26–28], minimum-acceptable benefit [28], relative importance weights [22, 24, 26], relative rank of AED attributes [22] and coefficient of the attributes [23–25]. Other formats were reported, such as additional seizure control that participants are willing to give up to improve units of adverse effects [25] and maximum acceptable incremental risk (%) per 1% increase in 12-month remission [23]. The statistical uncertainty associated with the estimates was reported using a 95% CI.

### Results of individual studies

This section summarizes which attributes are most important for people with epilepsy as reported in identified studies. Further details on the study aims and level of attributes are available in Table 1.

In Lloyd et al. [25], a pair of hypothetical AED profiles were described using five attributes related to adverse effects plus seizure control and cost (to estimate willingness to pay). The study presented the WTP for a unit improvement in each attribute level; for example, participants are willing to pay £2.67 per month per 1% reduction in the chance of hair loss. The study also presented the absolute WTP for different health states; for instance, respondents were willing to pay £709 per month for being seizure free with no adverse effects (the currency year is 2002). The study found that participants were willing to give up improvement in seizure control to avoid adverse effects. Segmented models showed that participants would be willing to pay 34%–42% of their income for being seizure free and experiencing no adverse effects on their health state and that women were willing to pay twice as much as men to avoid weight gain.

The study aim of Manjunath et al. [26] was to quantify the relative importance of 8 attributes of add-on AEDs. Seizure reduction was the most preferred outcome, with a mean relative importance of 10.0, followed by limitations due to long-term confusion or memory problems (8.6) and weight change (8.2). A treatment that provides freedom from seizures with no side effects and a single dosing was valued by respondents at $126.36 more than their current treatment (the currency year not reported is assumed to be 2012).

Ettinger et al. [22] compared patient and neurologist preferences regarding two hypothetical AEDs characterized by six attributes. Both patients and neurologists ranked seizure control as the most important attribute; however, seizure control had a significantly greater weighting in neurologists' decision-making than among patients (45% vs. 32%, *p* < 0.005). Patients placed more importance than neurologists on psychiatric adverse effects (19% vs. 15%, *p* < 0.05), diminished coordination and balance (16% vs. 10%, *p* < 0.05), and fatigue or diminished energy (13% vs. 11% m *p* < 0.05).

Holmes et al. [23] compared patient benefit-risk preferences between recently diagnosed patients and women of childbearing age. The results show that respondents had stronger preferences for reductions in the risk of AEs than improvements in 12-month seizure remission. In the recently diagnosed group, the maximum acceptable incremental risk of adverse effects for an AED that increased the 12-month seizure remission by 10% was 3.1% for depression, 3.0% for memory problems, and 2.5% for aggression. Women of childbearing age would accept an incremental risk of 5.6% for depression, 3.4% for memory problems and 2.0% for fetal abnormality for an AED that increases the 12-month seizure remission by 10%. The authors also applied the preference weights derived from discrete choice experiments to clinical trial data to estimate the expected utility of five alternative drugs.

Hua et al. [24] examined Chinese patients’ risk–benefit preferences and WTP for AED treatment. Seizure control was the most preferred treatment attribute (10.0; 95% CI 8.9–11.1), followed by the effects of AEDs on the fetus (8.9; 95% CI 7.7–10.1), the duration of side effects in the neuropsychic system (4.9; 95% CI 3.7–6.0), and adverse reactions of the digestive system (3.2; 95% CI 1.5–4.2). The participants were willing to spend ¥1,246 (95% CI, U 632–U 1,861) per month to ensure 100% seizure control and ¥ 1,112 (95% CI, U 586–U 1,658) to reduce the risk of the drug affecting the fetus by 3%. The currency year was not reported and was assumed to be 2020. Furthermore, patients who have the intention of getting pregnant have a high preference for lowering the effects on the fetus than patients who are not and value the effect of AEDs on the fetus more than the efficacy of AEDs.

Sinha et al. [28] determined patients acceptability of benefit-risk trade-offs in selecting treatment options for drug-resistant epilepsy, including open brain surgery, laser ablation or continued medications. The study findings show that respondents who were willing to undergo a procedure were willing to accept a markedly lower chance of freedom from seizures (23%) if they were able to undergo laser treatment as their first surgical option as opposed to 70% seizure freedom for open brain surgery. For 30-day mortality, the minimum acceptable benefit was 52% (95% CI 39–68%) for risk reduction from 1 to 0%. For the risk of long-term problems, the minimum acceptable benefit was 39% (95% CI 28–51%) for a risk reduction from 10 to 0%.

Rosenow et al. [27] explored patients' trade-offs between treatment efficacy and the risk of side effects of AEDs before and up to 14 days after treatment consultation and to elicit physicians’ preferences in selecting treatment for a specific patient after consultation with that patient. The most important attribute for patients before consultation was the chance of becoming seizure free with a mean conditional relative importance value of 10.0 (95% CI 7.8–12.2), followed by trouble thinking clearly (8.2, 95% CI 6.6–9.9) and personality changes (6.9, 95% CI 5.4–8.3). Patient preferences before and after treatment consultation were generally qualitatively similar. The mean conditional relative importance calculations showed that the most important attribute for physicians was the chance of becoming seizure free (10.0, 95% CI 8.6–11.4), followed by personality changes (7.5, 95% CI 6.2–8.9) and trouble thinking clearly (6.2, 95% CI 4.9–7.5).

## Discussion

In this review, we systematically searched three databases and identified seven studies that used the DCE technique to explore the preferences of people with epilepsy regarding antiepileptic medications. The findings indicate that people with epilepsy have strong preferences for improving seizure control, which was ranked as the top priority in all studies. Patients also have a strong preference for the reduction of adverse effects and may be willing to make a trade-off between improved seizure control and reduction of long-term side effects that may impact their quality of life. This is consistent with the findings of other reviews where efficacy and adverse effects are commonly investigated [15–17, 34] and rated highly important by the patients. It is worth noting that the diversity in attributes description and the levels chosen to define each attribute complicated the comparison of findings across studies, a shortcoming identified in previous reviews of DCEs [15, 16, 34].

Our reviews suggests that patients’ strength of preference varies between different patient subgroups which aligns with other reviews findings [17]. Two studies [22, 27] highlighted the difference in preferences between patients and physicians which have been reported in previous research [34, 35].

The review by Soekhai et al. [36] of DCEs in health economics updated prior reviews and identified 301 publications between 2013 and 2017. The majority of these DCEs are from the US and the UK, describe alternatives using 4–9 attributes and include 9–16 choices per individual. These characteristics are similar to the DCEs in epilepsy identified in our review.

The major source of identifying attributes and levels was literature reviews which corresponds with the findings of previous systematic reviews [15, 16, 34]; however, included studies did not report on the comprehensiveness of the literature reviews to ensure that the best available evidence was used. The use of a qualitative approach to identify attributes, assign levels, assess the understandability of text and explain and evaluate layout and length is recommended and common in discrete choice experimental design [37, 38]. In our review, the authors reported that attributes were identified or selected through consultations with experts or patients without explaining the characteristics of these consultations or the issues discussed. Similarly, the paucity in reporting the qualitative component of the study in detail has been observed in a systematic review of qualitative research methods in DCEs [39].

The included studies described the level of side effects of antiepileptic drugs through severity and likelihood of these side effects. However, as Vass and Payne [38] explained, “Risk is a multifaceted concept.” Many components of risk communication, such as severity, duration, irreversibility, certainty and the baseline level of risk, if left unexplained, are subsequently inferred by respondents using their own values, leading to compromised and biased estimates [38]. The methodological issues regarding the use of DCE in benefit-risk assessment represent an area that requires further investigation and guidance for researchers [38].

This review found great heterogeneity in terms of choice of experimental design and model estimation. The choice of experiment design depends on many factors including number of attributes, number and types of levels and interaction between attributes. The published ISPOR reports [10, 40] on discrete choice experiments provide guidance without endorsing any specific methodological approach, however; researchers are expected to explain and justify the chosen approach. Similar to previous reviews [15, 16, 34, 40], our study demonstrated that justification of experimental design, evaluation of experimental design properties and description of model estimation were poorly reported in discrete choice experiments.

An introductory section that explains attributes, levels and shows a practice version of the choice tasks is recommended [10]; however, this aspect was unclear in many of the included studies. The use of interactive tools for this purpose, especially online tools, provides respondents with the capability, opportunity and motivation to complete a DCE [38]. The interviewer-led administration of the DCE survey was an underused approach in included studies which is in line with the findings of other reviews [15, 16, 34, 36]. Compared with self-administered questionnaire, interviewer-led administration may improve the quality of data collected as the interviewer can explain the tasks and answer a respondent’s questions [10].

### Implications for future research

The number of identified studies is limited. There is a need for more research in this area. Future research should build on the findings of this review with regard to important elements of study design that have received little attention in the published literature. The first area is the identification and selection of attributes to describe alternative choices. We recommend more details on the purpose of the qualitative process, e.g., to identify or validate attributes and levels, order attributes, classify level ranges or test the complexity of the survey; the qualitative approaches used, e.g., structured or semistructured interviews, focus groups, think aloud exercises, or observations of patients’ decision-making processes; and approaches used to analyze qualitative data. Helter et al. [41] suggested four stages for attribute development: raw data collection; data reduction; removing inappropriate attributes; and wording using different qualitative and alternative methods for each stage. This framework can be used to describe attribute development in future research. Secondary areas that require attention include justification for the choice of experimental design used to create tasks and the model estimations approach to analyze data.

The focus of available evidence was on efficacy and side effects, and we recommend further qualitative research to explore other attributes that might also be important for patients with epilepsy. People with epilepsy have many concerns about living with epilepsy in addition to seizure control and medication side effects [42] that may influence their preferences for interventions.

New studies should also examine questions not explored by the published research, which could be achieved, for instance, by investigating underrepresented patient groups such as children, adolescents or elderly individuals, the preference for discontinuation of treatment and the impact of patient characteristics on preferences. Indeed, understanding aspects that may explain preference heterogeneity, such as patient characteristics, is one of the methodological priorities for patient preference research in general [43].

The main strength of this review is the systematic search for evidence and quality appraisal of the included studies. There is a chance that we missed some studies published in journals not indexed in the databases we searched. Another limitation of our method is that data were extracted from published manuscripts without attempts to obtain or confirm data from the investigators. In our review, we excluded studies measuring stated preference using direct elicitation of monetary values of an intervention, such as willingness-to-pay methods, and preference-based studies, such as time-trade-offs or standard gambles, referred to as preference-based but not considered stated preferences. The reason for their exclusion was to review studies with homogenous methodologies, as excluded studies differ in their methodologies, results use and implications. Future reviews may examine such types of studies.

## Conclusions

The use of DCEs in measuring patients’ preferences for epilepsy treatment is accumulating. However, inadequate reporting of methodological details may reduce decision-makers’ confidence in the findings. Suggestions for future research are provided.

## Supplementary Information

**Additional file 1.**

**Additional file 2.**

## Data Availability

Data sharing is not applicable to this systematic review, as no primary data were generated during the current study. Data supporting the conclusions of this article are included within the article and in additional files.

## Abbreviations

AEDs: Antiepileptic drugs
DCEs: Discrete choice experiments
PRISMA: Preferred Reporting Items for Systematic Reviews and Meta-analyses
QALYs: Quality-adjusted life years

## References

[1] Thijs RD, Surges R, O’Brien TJ, SJ. Epilepsy in adults. Lancet. 2919;16(393):689–701. 10.1016/S0140-6736(18)32596-0 30686584

[2] Krahn M NG. The next step in guideline development: incorporating patient preferences. JAMA. 2008;300(4):436. 10.1001/jama.300.4.436. 10.1001/jama.300.4.436 18647988

[3] Nygaard A, Halvorsrud L, Linnerud S, Grov EK, Bergland A (2019). The James Lind Alliance process approach: Scoping review. BMJ Open.

[4] Kim C, Armstrong MJ, Berta WB, Gagliardi AR (2020). How to identify, incorporate and report patient preferences in clinical guidelines: a scoping review. Heal Expect.

[5] Blackwood J, Armstrong MJ, Schaefer C, Graham ID, Knaapen L, Straus SE (2020). How do guideline developers identify, incorporate and report patient preferences? An international cross-sectional survey. BMC Health Serv Res.

[6] Marsh K, van Til JA, Molsen-David E, Juhnke C, Hawken N, Oehrlein EM (2020). Health Preference Research in Europe: A Review of Its Use in Marketing Authorization, Reimbursement, and Pricing Decisions—Report of the ISPOR Stated Preference Research Special Interest Group. Value Heal.

[7] Epilepsies in children, young people and adults (NICE guideline NG217). National Institute for Health and Care Excellence; 2019. 1–11 p. Available from: https://www.nice.org.uk/guidance/indevelopment/gid-ng10112 35700280

[8] Janssens R, Huys I, Van Overbeeke E, Whichello C, Harding S, Kübler J (2019). Opportunities and challenges for the inclusion of patient preferences in the medical product life cycle: A systematic review. BMC Med Inform Decis Mak.

[9] Losi S, Berra CCF, Fornengo R, Pitocco D, Biricolti G, Federici MO (2021). The role of patient preferences in adherence to treatment in chronic disease: a narrative review. Drug Target Insights.

[10] Bridges JFP, Hauber AB, Marshall D, Lloyd A, Prosser LA, Regier DA (2011). Conjoint analysis applications in health - A checklist: a report of the ISPOR good research practices for conjoint analysis task force. Value Heal.

[11] Ryan M, Scott DA, Reeves C, Bate A, van Teijlingen ER, Russell EM, Napper MRC (2001). Eliciting public preferences for healthcare: a systematic review of techniques. Heal Technol Assess.

[12] Soekhai V, Whichello C, Levitan B, Veldwijk J, Pinto CA, Donkers B (2019). Methods for exploring and eliciting patient preferences in the medical product lifecycle: a literature review. Drug Discov Today.

[13] Louviere JJ, Flynn TN, Carson RT (2010). Discrete choice experiments are not conjoint analysis. J Choice Model.

[14] Louviere JJ, Lancsar E (2009). Choice experiments in health: the good, the bad, the ugly and toward a brighter future. Heal Econ Policy Law.

[15] Tünneßen M, Hiligsmann M, Stock S, Vennedey V (2020). Patients’ preferences for the treatment of anxiety and depressive disorders: a systematic review of discrete choice experiments. J Med Econ.

[16] Bien DR, Danner M, Vennedey V, Civello D, Evers SM, Hiligsmann M (2017). Patients’ preferences for outcome, process and cost attributes in cancer treatment: a systematic review of discrete choice experiments. Patient.

[17] Collacott H, Zhang D, Heidenreich S, Tervonen T (2022). A systematic and critical review of discrete choice experiments in asthma and chronic obstructive pulmonary disease. Patient.

[18] Page MJ, McKenzie JE, Bossuyt PM, Boutron I, Hoffmann TC, Mulrow CD (2021). The PRISMA 2020 statement: an updated guideline for reporting systematic reviews. PLoS Med.

[19] Van Hoorn R, Kievit W, Booth A, Mozygemba K, Lysdahl KB, Refolo P (2016). The development of PubMed search strategies for patient preferences for treatment outcomes. BMC Med Res Methodol.

[20] Selva A, Solà I, Zhang Y, Pardo-Hernandez H, Haynes RB, Martínez García L (2017). Development and use of a content search strategy for retrieving studies on patients’ views and preferences. Health Qual Life Outcomes.

[21] Joy SM, Little E, Maruthur NM, Purnell TS, Bridges JFP (2013). Patient preferences for the treatment of type 2 diabetes: A scoping review. Pharmacoeconomics.

[22] Ettinger AB, Carter JA, Rajagopalan K (2018). Patient versus neurologist preferences: a discrete choice experiment for antiepileptic drug therapies. Epilepsy Behav.

[23] Holmes EAF, Plumpton C, Baker GA, Jacoby A, Ring A, Williamson P (2019). Patient-focused drug development methods for benefit-risk assessments: a case study using a discrete choice experiment for antiepileptic drugs. Clin Pharmacol Ther.

[24] Hua Y, Zhu Z, Li X, Gong J, Ding S, Lin J, et al. Patient preference for antiepileptic drugs treatment in China: evidence from the discrete choice experiment. Front Neurol. 2020;11:602481. 10.3389/fneur.2020.602481. 10.3389/fneur.2020.602481 PMC7744628 33343502

[25] Lloyd A, McIntosh E, Price M (2005). The importance of drug adverse effects compared with seizure control for people with epilepsy: a discrete choice experiment. Pharmacoeconomics.

[26] Manjunath R, Yang JC, Ettinger AB (2012). Patients’ preferences for treatment outcomes of add-on antiepileptic drugs: a conjoint analysis. Epilepsy Behav..

[27] Rosenow F, Winter Y, Leunikava I, Brunnert M, Joeres L, Sutphin J, et al. Relative importance of clinical outcomes and safety risks of antiseizure medication monotherapy for patients and physicians: Discrete choice experiment eliciting preferences in real-world study “VOTE.” Epilepsia. 2022;63(2):451–62. 10.1111/epi.17137 34921391

[28] Sinha SR, Yang JC, Wallace MJ, Grover K, Johnson FR, Reed SD. Patient preferences pertaining to treatment options for drug-resistant focal epilepsy. Epilepsy Behav. 2022;127:108529. 10.1016/j.yebeh.2021.108529. 10.1016/j.yebeh.2021.108529 35016055

[29] Perry MS, Swint C, Hawley J, Kohler S, Blake S, Rask K (2011). Caregiver measures for seizure control, efficacy, and tolerability of antiepileptic drugs for childhood epilepsy: results of a preference survey. Epilepsy Behav.

[30] Atkinson-Clark E, Charokopou M, Van Osselaer N, Hiligsmann M (2018). A discrete-choice experiment to elicit preferences of patients with epilepsy for self-management programs. Epilepsy Behav.

[31] Nicolet A, Van Asselt ADI, Vermeulen KM, Krabbe PFM (2020). Value judgment of new medical treatments: societal and patient perspectives to inform priority setting in the Netherlands. PLoS One.

[32] Apantaku G, Aguiar M, Kaal KJ, McDonald PJ, Connolly MB, Hrincu V (2022). Understanding attributes that influence physician and caregiver decisions about neurotechnology for pediatric drug-resistant epilepsy: a formative qualitative study to support the development of a discrete choice experiment. Patient.

[33] Shingler SL, Howard C, LA Johnston K (2013). Patient preferences for therapies in Epilepsy. Value Heal.

[34] Sain N, Willems D, Charokopou M, Hiligsmann M (2020). The importance of understanding patient and physician preferences for psoriasis treatment characteristics: a systematic review of discrete-choice experiments. Curr Med Res Opin.

[35] Mühlbacher AC, Juhnke C (2013). Patient preferences versus physicians’ judgement: does it make a difference in healthcare decision making?. Appl Health Econ Health Policy.

[36] Soekhai V, de Bekker-Grob EW, Ellis AR, Vass CM (2019). Discrete Choice Experiments in Health Economics: Past Present and Future. Pharmacoeconomics..

[37] Kløjgaard ME, Bech M, Søgaard R (2012). Designing a stated choice experiment: The value of a qualitative process. J Choice Model.

[38] Vass CM, Payne K (2017). Using discrete choice experiments to inform the benefit-risk assessment of medicines: are we ready yet?. Pharmacoeconomics.

[39] Vass C, Rigby D, Payne K (2017). The role of qualitative research methods in discrete choice experiments: a systematic review and survey of authors. Med Decis Mak.

[40] Johnson FR, Lancsar E, Marshall D, Kilambi V, Mühlbacher A, Regier DA (2013). Constructing experimental designs for discrete-choice experiments: report of the ISPOR conjoint analysis experimental design good research practices task force. Value Heal.

[41] Helter TM, Boehler CEH (2016). Developing attributes for discrete choice experiments in health: a systematic literature review and case study of alcohol misuse interventions. J Subst Use.

[42] Sander JW (2005). Ultimate success in epilepsy - The patient’s perspective. Eur J Neurol Suppl.

[43] Smith IP, DiSantostefano RL, de Bekker-Grob EW, Levitan B, Berlin C, Veldwijk J (2021). Methodological priorities for patient preferences research: stakeholder input to the PREFER public-private project. Patient.

