# Supplementary material for: Patient preferences for epilepsy treatment: a systematic review of discrete choice experimental studies
Source: Health Econ Rev. 2023 Mar 18;13:17. doi: 10.1186/s13561-023-00431-0 (PMC10024410; doi:10.1186/s13561-023-00431-0)
Supplement: Supplementary file 1 — Additional file 1. [file 13561_2023_431_MOESM1_ESM.docx]

**Appendix 1 Search strategy for each database**

| **Database** | Search | **Number of hits** |
| --- | --- | --- |
| **Pubmed**  **16^th^ Feb 2022** | Discrete Choice Experiment[Title/Abstract] OR Discrete Choice Experiments[Title/Abstract] OR Discrete Choice modeling[Title/Abstract] OR Discrete Choice modelling[Title/Abstract] OR Discrete choice conjoint experiment[Title/Abstract] OR Stated preference[Title/Abstract] OR Conjoint analysis[Title/Abstract] OR Conjoint choice experiments[Title/Abstract] OR Conjoint choice experiment[Title/Abstract] OR Conjoint measurement[Title/Abstract] OR Conjoint studies[Title/Abstract] OR Paired comparisons[Title/Abstract] OR Pairwise choices[Title/Abstract]  OR Part worth utilities[Title/Abstract] OR Functional measurement[Title/Abstract] OR Paired comparison[MeSH Terms] OR Patient Preference[MeSH Terms] AND ("epilepsy"[Title/Abstract] OR "epilepsy"[MeSH Terms]) | 72 |
| **Web of Science**  **16^th^ Feb 2022** | ((((TS=(Stated preference )) OR TS=(Discrete Choice Experiments )) OR TS=(Conjoint analysis )) OR TS=(Patient Preference)) AND TS=(epilepsy ) | 358 |
| **SCOUPS**  **8^th^ April 2022** | ( TITLE-ABS-KEY ( discrete  AND choice  AND experiment )  OR  TITLE-ABS-KEY ( conjoint  AND analysis )  OR  TITLE-ABS-KEY ( patient  AND preference )  OR  TITLE-ABS-KEY ( stated  AND preference )  AND  TITLE-ABS-KEY ( epilepsy ) ) | 531 |
