# Supplementary material for: Patient preferences for epilepsy treatment: a systematic review of discrete choice experimental studies
Source: Health Econ Rev. 2023 Mar 18;13:17. doi: 10.1186/s13561-023-00431-0 (PMC10024410; doi:10.1186/s13561-023-00431-0)
Supplement: Supplementary file 2 — Additional file 2. [file 13561_2023_431_MOESM2_ESM.docx]

**Appendix 2 Risk of bias assessment**

PREFS (Purpose, Respondents, Explanation, Findings, Significance) checklist

|  | **Lloyd**  **2005** | **Manjunath**  **2012** | **Ettinger**  **2018** | **Holmes**  **2018** | **Hua**  **2020** | **Sinha**  **2021** | **Rosenow**  **2022** |
| --- | --- | --- | --- | --- | --- | --- | --- |
| Purpose: Is the purpose of the study in relation to preferences clearly stated? | Yes | Yes | Yes | Yes | Yes | Yes | Yes |
| (2) Respondents: Are the responders similar to the non-responders? | Unclear | Unclear | Unclear | Unclear | Unclear | Unclear | Unclear |
| (3) Explanation: Are methods of assessing preferences clearly explained? | Yes | Yes | Yes | Yes | Yes | Yes | Yes |
| (4) Findings: Were all respondents included in the reported findings and analysis of preference results? | Yes | Yes | Yes | Yes | Yes | Yes | No |
| (5) Significance: Were significance tests used to assess the preference results? | Yes | Yes | Yes | Yes | Yes | Yes | Yes |

ISPOR Conjoint Analysis Applications in Health Checklist

| **Checklist/Article** | **Lloyd, 2005** | **Manjunath,2012** | **Ettinger, 2018** | **Holmes, 2018** | **Hua, 2020** | **Sinha,2021** | **Rosenow,2022** |
| --- | --- | --- | --- | --- | --- | --- | --- |
| **1. Was a well-defined research question stated and is conjoint analysis an appropriate method for answering it?** | | | | | | | |
| 1.1 Were a well-defined research question and a testable hypothesis articulated? | Y | Y | Y | Y | Y | Y | Y |
| 1.2 Was the study perspective described, and was the study placed in a particular decision-making or policy context? | Y | Y | Y | Y | Y | Y | Y |
| 1.3 What is the rationale for using conjoint analysis to answer the research question? | Y | Y | Y | Y | Y | Y | Y |
| **2. Was the choice of attributes and levels supported by evidence?** | | | | | | | |
| 2.1 Was attribute identification supported by evidence (literature reviews, focus groups, or other scientific methods)? | Y | Y | Y | Y | Y | Y | N |
| 2.2 Was attribute selection justified and consistent with theory? | Y | N | Y | Y | Y | N | Y |
| 2.3 Was level selection for each attribute justified by the evidence and consistent with the study perspective and hypothesis? | Y | N | Y | Y | Y | N | Y |
| **3. Was the construction of tasks appropriate?** | | | | | | | |
| 3.1 Was the number of attributes in each conjoint task justified (that is, full or partial profile)? | Y | Y | Y | Y | Y | Y | Y |
| 3.2 Was the number of profiles in each conjoint task justified? | Y | Y | Y | Y | Y | Y | Y |
| 3.3 Was (should) an opt-out or a status-quo alternative (be) included? | N | Y | N | N | N | Y | N |
| **4. Was the choice of experimental design justified and evaluated?** | | | | | | | |
| 4.1 Was the choice of experimental design justified? Were alternative experimental designs considered? | Y | N | Y | N | Y | Y | N |
| 4.2 Were the properties of the experimental design evaluated? | Y | N | Y | N | N | Y | N |
| 4.3 Was the number of conjoint tasks included in the data-collection instrument appropriate? | Y | Y | N | Y | Y | Y | Y |
| **5. Were preferences elicited appropriately, given the research question?** | | | | | | | |
| 5.1 Was there sufficient motivation and explanation of conjoint tasks? | Y | N | N | Y | N | Y | N |
| 5.2 Was an appropriate elicitation format (that is, rating, ranking, or choice) used? Did (should) the elicitation format allow for indifference? | Y | Y | Y | Y | Y | Y | Y |
| 5.3 In addition to preference elicitation, did the conjoint tasks include other qualifying questions (for example, strength of preference, confidence in response and other methods?) | N | N | N | N | N | N | N |
| **6. Was the data collection instrument designed appropriately?** | | | | | | | |
| 6.1 Was appropriate respondent information collected (such as sociodemographic, attitudinal, health history or status, and treatment experience)? | Y | Y | Y | Y | Y | Y | Y |
| 6.2 Were the attributes and levels defined, and was any contextual information provided? | Y | Y | N | Y | Y | Y | Y |
| 6.3 Was the level of burden of the data-collection instrument appropriate? Were respondents encouraged and motivated? | Y | Y | Y | Y | Y | Y | Y |
| **7. Was the data-collection plan appropriate?** | | | | | | | |
| 7.1 Was the sampling strategy justified (for example, sample size, stratification, and recruitment)? | N | Y | Y | Y | Y | N | N |
| 7.2 Was the mode of administration justified and appropriate (for example, face-to-face, pen-and-paper, web-based)? | Y | Y | Y | Y | Y | Y | Y |
| 7.3 Were ethical considerations addressed (for example, recruitment, information and/or consent, compensation)? | N | Y | Y | Y | Y | Y | Y |
| **8. Were statistical analyses and model estimations appropriate?** | | | | | | | |
| 8.1 Were respondent characteristics examined and tested? | Y | Y | Y | Y | Y | Y | Y |
| 8.2 Was the quality of the responses examined (for example, rationality, validity, reliability)? | Y | Y | N | Y | Y | Y | N |
| 8.3 Was model estimation conducted appropriately? Were issues of clustering and subgroups handled appropriately? | Y | Y | Y | Y | Y | Y | Y |
| **9. Were the results and conclusions valid?** | | | | | | | |
| 9.1 Did study results reflect testable hypotheses and account for statistical uncertainty? | Y | Y | Y | Y | Y | Y | Y |
| 9.2 Were study conclusions supported by the evidence and compared with existing findings in the literature? | Y | Y | Y | Y | Y | Y | Y |
| 9.3 Were study limitations and generalizability adequately discussed? | Y | Y | Y | Y | Y | Y | Y |
| **10. Was the study presentation clear, concise, and complete?** | | | | | | | |
| 10.1 Was study importance and research context adequately motivated? | Y | Y | Y | Y | Y | Y | Y |
| 10.2 Were the study data-collection instrument and methods described? | Y | Y | Y | Y | Y | Y | Y |
| 10.3 Were the study implications clearly stated and understandable to a wide audience? | Y | Y | Y | Y | Y | Y | Y |

Y=Yes, N=No
